# Pathogenicity and virulence of *Candida auris*

**DOI:** 10.1080/21505594.2026.2707827

**Published:** 2026-07-31

**Authors:** Trinh Phan-Canh, Saskia Seiser, Manju Chauhan, Karl Kuchler, Neeraj Chauhan, Adelheid Elbe-Bürger

**Affiliations:** a Max Perutz Labs Vienna, Vienna Biocenter Campus (VBC), Vienna, Austria; b Center for Medical Biochemistry, Medical University of Vienna, Vienna, Austria; c Department of Dermatology, Medical University of Vienna, Vienna, Austria; d Center for Discovery and Innovation, Hackensack Meridian Health, Nutley, NJ, USA

**Keywords:** *Candida auris*, *Candidozyma auris*, virulence, skin tropism, host-pathogen interaction, immune response

## Abstract

*Candida auris (Candidozyma auris)* has emerged as a multidrug‑resistant human fungal pathogen that causes infections of high morbidity and mortality. Notably, it exhibits a unique ability to grow and persist on human skin, thus leading to efficient transmission through skin-to-skin contact. As a result, *C. auris* poses a significant risk of outbreaks in healthcare settings, especially in nursing homes that care for elderly patients. Most concerning, *C. auris* clinical isolates demonstrate widespread and, in some cases, untreatable resistance to all antifungal drug classes, including azoles, polyenes (amphotericin B), and echinocandins. Consequently, invasive *C. auris* infections cause high mortality rates (30–60%) even with antifungal therapy. Here, we provide a comprehensive overview of candidiasis caused by *C. auris*, discussing both host and pathogen determinants of skin colonization, as well as key challenges associated with preventing dissemination and management of disseminated fungal infections.

## Introduction

### The origin and evolution of *Candida auris*

The initial reports of *Candida auris* (recently reclassified as *Candidozyma auris*, although this nomenclature remains under discussion [1]), from Japan in 2009 and South Korea in 2011 [2], did not suggest that this pathogen had substantial potential for transmission. Since its initial identification, *C. auris* has spread extensively worldwide, leading to multiple outbreaks, with over 84,000 reported cases of colonization and infection as of December 2025 [3,4]. *C. auris* has now been reported in over 80 countries across all six World Health Organization (WHO) regions, and is responsible for prolonged outbreaks in hospital settings [3–7]. A key question has been whether *C. auris* emerged recently as a pathogen or whether it was historically present but was misidentified as other *Candida* spp. due to inaccurate diagnostic tests routinely used in medical mycology. Large-scale surveillance programs such as the SENTRY Antifungal Surveillance Program and sentinel candidemia surveillance at multiple sites in the US failed to detect *C. auris* prior to 2009 [8,9]. The earliest known retrospective *C. auris* isolates date back to 1996 in South Korea and 1997 in Japan [3]. Therefore, *C. auris* likely evolved into a human fungal pathogen in the past decades.

A population genomic analysis of over 300 isolates from 19 countries across six continents estimated that *C. auris* originated from a common ancestor over the past centuries, with outbreak-causing clusters from clades I, III, and IV emerging some decades ago [10]. Whole genome sequencing suggests that most clinical isolates form four major clades, including South Asian (I), East Asian (II), African (III) and South American (IV), predominant in the corresponding geographic regions. Although clades are largely region-specific, global travel and trade have been promoting the spreading of *C. auris* [11]. Recently, clade V was reported in Iran, and clade VI in Singapore and Bangladesh [12–14], where clade I is otherwise prevailing. Although clade V and VI isolates appear largely susceptible to azoles and echinocandins, they likely pose the same outbreak and nosocomial transmission risks as other clades, with comparable capacity to persist on medical devices and environmental surfaces [12–14]. Further genomic and clinical surveillance is nonetheless essential before drawing firm conclusions about these emerging clades. New clades typically carry genomic deletions, inversions, translocations, and thousands of single nucleotide polymorphisms (SNPs) [15,16]. For instance, clade II strains carry deletions in sub-telomeric regions, ablating more than 10 genes encoding putative adhesins and glycosylphosphatidylinositol (GPI)-anchored proteins [15]. This implies fitness defects and partially explain their hyper-susceptibility to cell wall stress agents such as Calcofluor White and Congo Red [17], disinfectants [18] and antifungal drugs [11].

In recent years, *C. auris* has been found in various ecological niches [19–21]. The discovery of *C. auris* on an isolated salt marshlands on the South Andaman islands suggests an origin in marine ecosystems, which corresponds to its intrinsic halotolerance [21]. Interestingly, the isolate VPCI/E/AN/176/20 from South Andaman exhibits lower thermotolerance and lower antifungal tolerance when compared to other clinical *C. auris* strains, implying that global warming and the overuse of agricultural fungicides, could drive the adaptive acquisition of multidrug resistance (MDR) traits as well as stress tolerance [22]. Noteworthy, relatively few SNPs distinguish the Andaman strain from nine clinical patient isolates (67–153 SNPs), and most SNPs are in fact located in non-coding regions. However, certain mutations in coding regions, including Y132F in *ERG11*, A583S in *TAC1B*, L351M in *ERG7*, and K719N in *STE6* [21], may explain altered antifungal susceptibilities of environmental strains. Interestingly enough, other ecological niches for *C. auris* such as apples [23] have been reported, highlighting potential roles of agricultural fungicides in MDR development. Finally, animals such as chickens [24], snakes [20] and dogs [19,25] are additional potential reservoirs for *C. auris*. All in all, *C. auris* is a high-priority fungal pathogen whose emergence may be directly linked to climate change potentially facilitating host range expansion to humans [26,27].

*C. auris* has a haploid genome with a size of approximately 12.1–12.7 Mb, encoding around 5,200–5,600 proteins, organized across seven chromosomes [15,16], with a GC-content of about 45%. Furthermore, *C. auris* belongs to the CTG clade of *Candida* species [16], which is distinguished by a unique deviation from the universal genetic code [28]. In these fungi, the CTG codon is translated as serine rather than the standard leucine [29,30]. Despite being part of this clade, *C. auris* is highly divergent from other pathogenic *Candida* spp., including *C. albicans*, *C. parapsilosis*, and *C. tropicalis*. Although mating or a sexual cycle has not been observed in *C. auris*, most mating and meiosis-related genes are conserved. The *MTL***a** or *MTL*α mating loci are clade-specific, whereby clades I, IV, and V isolates are *MTL***a**, while clades II, III, and VI are *MTL*α [13,16]. This clade-specific mating type is consistent with clonal divergence and asexual reproduction within clades. Although evidence is as yet unavailable, mating could potentially occur between competent clade strains, especially given the presence of strains from different clades in the same hospital [31]. Intra-clade differences are typically marked by a few dozen SNPs, whereas inter-clade variations involve thousands of SNPs [13,16,31], although mainly in non-coding regions. This suggests that genome features alone cannot fully explain the extensive intra-clade heterogeneity related to virulence and pronounced antifungal resistance [32], particularly the exceptionally high rate of amphotericin B resistance in *C. auris* isolates when compared to other pathogenic yeast species.

### Epidemiology and infection control of *C.* *auris*

The prevalence of candidemia caused by *C. auris* varies geographically but has been ever-increasing. In the US, clinical cases of *C. auris* tripled from 476 in 2019 to 1,471 in 2021, with echinocandin-resistant cases nearly three times higher in 2021 than in the previous two years [6]. In 2025, over 7000 *C. auris* cases were reported in 27 US states [33]. In Europe, 4000 cases were reported between 2012 and 2023, including 1346 cases reported in 2023 alone [34]. National surveillance in South Africa reported 1,579 cases from 2012 to 2016, out of which 794 occurred in just two years from 2016 to 2017 [35,36]. In Asia, *C. auris* was first reported in Japan [37], Korea [2,38], and India [39,40], then in China [41], the Middle East [42] and Southeast Asia [43–46], with a novel clade recently identified in Singapore and Bangladesh [12,13]; the burden is now substantial in several regions, accounting for roughly 5–30% of candidemia in some Indian ICUs and rising sharply in China after 2022, partly linked to relaxed COVID-19 controls measures in late 2022 [4,47]. Hospital outbreaks are on the rise worldwide [48]. For instance, large outbreaks were documented in nearly 100 hospitals in South Africa, where *C. auris* now accounts for 10% of all candidemia cases [36]. A 27-center study [49] in India shows that *C. auris* was responsible for 5.3% of invasive candidemia cases, with a single hospital reporting a remarkable prevalence of 39% [50]. The rapid spread of *C. auris* outbreaks poses a significant global health threat, prompting the WHO to designate this emerging fungal pathogen as a critical priority for research and drug development [51].

The overall mortality in patients with disseminated *C. auris* infections ranges from 29% to 62% [48]. Of note, prolonged ICUs hospitalization (>2 weeks) was observed in roughly 70% of reported *C. auris* candidemia cases [48]. Risk factors for *C. auris* infections include renal dysfunction, extended stays in cardiothoracic units or in ICUs for more than 10–15 days. ICU patients are at increased risk due to factors such as mechanical ventilation, central venous catheters (CVC), parenteral nutrition, and enhanced sepsis susceptibility, which is often further exacerbated by superinfections with bacteria or viruses [52]. Additionally, prior administration of antifungal drugs such as triazoles or echinocandins within 30 days of a *C. auris* candidemia diagnosis, constitutes a significant risk factor, suggesting that prophylactic clinical antifungal use requires a careful consideration [48]. Management and prevention of *C. auris* infections are challenging due to its pronounced adherence to and persistence on abiotic plastic surfaces as well as biotic surfaces such as human skin. The extreme skin-tropism is responsible for the easy person-to-person transmission among healthcare professionals and patients through skin contacts. Remarkably, *C. auris* can persist on patient skin for up to eight months even after discharge from hospitals [53], while in-patients in healthcare facilities often remain continuously colonized [54]. *C. auris* tends to predominate on areas such as the perianal skin, axilla, nares, inguinal crease, palms, fingertips, and toe webs. Interestingly, these anatomical regions are frequently enriched by skin microbiome components such as antibiotic-resistant ESKAPE bacterial microbes, including *Proteobacteria* such as *Proteus mirabilis, Klebsiella pneumoniae, Providencia stuartii*, and *Pseudomonas aeruginosa* [55,56]. Our own recent findings suggest that the co-enrichment of *C. auris* with urease-positive bacteria (*P. mirabilis* and *K. pneumoniae*) on skin tissues can facilitate the scavenging of CO_2_ by *C. auris*, since the urease pathway yields CO_2_ by breaking down urea [57]. Interestingly, several common skin commensals, including *Staphylococcus hominis*, *Corynebacterium tuberculostearicum*, *Staphylococcus epidermidis*, *Staphylococcus caprae*, and *Corynebacterium striatum*, are preferentially detected in skin samples lacking *C. auris* [55,58].

Although more than 40 anti-septic fungicidal products are registered with the US Environmental Protection Agency (EPA) [59] as effective disinfectants for efficient hygiene measures to eradicate *C. auris*, most evidence comes from *in vitro* studies [18,60–63]. Their efficacy in skin decolonization *in vivo* remains uncertain. The quaternary ammonium compound chlorhexidine gluconate (CHG), commonly used for skin bathing and washing in clinical settings, shows inconsistent results in removing *C. auris* from skin [64,65]. Of note, combining 2% CHG with 70% isopropyl alcohol yields greater effectiveness in reducing *C. auris* when applied for two minutes when compared to CHG alone [65]. *In vitro* data suggest that wash mitts impregnated with anti-septic octenidine dihydrochloride were more effective than CHG [60]. Significant reduction of *C. auris* on the skin is accomplished with CHG concentrations exceeding ≥625 µg/ml [55]. Notably, this concentration is about 20–40 times higher than the *in vitro* minimum inhibitory concentrations of 16–32 µg/ml CHG against *C. auris* [55]. *C. auris* also colonizes skin invaginations and hair follicles, limiting the efficacy of disinfectants. Recently, we evaluated commercial octenidine-based antiseptics, which efficiently eradicate biofilms *in vitro* and *C. auris* colonization on human skin [66]. Therefore, effective skin decolonization of *C. auris* may require improvements concerning active ingredients, but also in pharmaceutical formulations to achieve quantitative disinfection.

Regarding environmental disinfection, several guidelines have been issued by various health organizations [67–69]. The most up-to-date recommendations are available on the US CDC website, which includes a list of EPA-recommended products and practical guidelines [69]. Many disinfection agents, including sodium hypochlorite, hydrogen peroxide, ethanol, and sodium dodecyl sulfate, have been tested against various *Candida* spp. [70,71]. Among these, 1% sodium hypochlorite was most effective against all tested *Candida* spp. growing in planktonic morphotypes or in biofilms [71]. For *C. auris*, the efficacy of chlorine-based agents was first demonstrated during the 2015 hospital outbreak in the United Kingdom, where a chlorine-based product was used at a 1000ppm concentration [5]. The recent detection of *C. auris* in wastewater in Nevada highlights the urgent need for robust environmental surveillance but also monitoring and testing disinfectant efficiency following wastewater treatment procedures [72,73].

### MDR burden and prevalence

Clinical MDR has been one of the major challenges in managing *C. auris* infections, since antifungal resistances are decisive factors governing clinical outcomes, particularly in cases of recurrent candidiasis [74]. Notably, about 90% of clinical isolates exhibit a high intrinsic resistance to most azoles, 30–60% are resistant to amphotericin B, and up to 10% are resistant to echinocandins [8,39,74]. Although 5-flucytosine (5FC) is rarely used in clinical settings anymore, resistance to this traditional drug occurs at extreme rates as high as 14% for clade I strains from India [39]. The first-line preemptive therapy for *C. auris* infections are echinocandins [75]. However, echinocandins have significant limitations, including the lack of oral formulations, high costs, and poor bioavailability especially in the urinary tract or central nervous system [74]. Furthermore, resistance often evolves during clinical treatment through *de novo* mutations, leading to therapy failures, particularly in cases of recurrent infections or prolonged hospital stays [76,77].

Strikingly, more than 90% of clinical *C. auris* isolates are resistant to at least one antifungal drug, around 30% are resistant to at least two classes, and some strains exhibit resistance to all three major anti-fungal drugs [78]. This underscores *C. auris* as an exceptional multidrug-resistant pathogen, a serious clinical phenotype not commonly observed in *C. albicans*. Although six clades of *C. auris* have been identified to date [12,13], invasive MDR infections are primarily associated with strains from clades I, III, and IV. In contrast, clade II strains remain sensitive to antifungals and are usually associated with ear infections rather than invasive diseases [79]. Key mechanisms of antifungal MDR in *C. auris* have been extensively discussed in several other reviews [11,80,81].

## Phenotypic plasticity and morphogenesis

Several fungal pathogens, including *Candida*, *Histoplasma*, *Coccioides* and *Aspergillus* spp., employ morphogenetic alterations as a strategy to adapt to different environmental conditions or stressful host defense-related stress encountered in different niches [82–84]. Likewise, *C. auris* has the ability to undergo certain morphogenetic changes under host-relevant conditions [85]. Although underlying mechanisms remain somewhat enigmatic, several reports identify distinct morphotypes of *C. auris* [86–89]. Based on current knowledge, morphogenesis is reflected by phenomena such as the yeast *vs* filamentation transition [88,89]; aggregated *vs* non-aggregated forms [90,91] and reversible phenotypic switching [86,92,93] yielding distinct morphotypes.

### Filamentation

Filamentation is a well-characterized virulence trait in *C. albicans*, playing a crucial role in breaching epithelial barriers before organ dissemination in invasive infections [82,83]. Virulence factors in *C. albicans* often differ significantly between the yeast and hyphal growth forms [83]. Although filamentation does not appear as a dominant trait in *C. auris*, it does occur at low frequencies in liquid culture and can be induced under specific conditions [89]. However, the frequency of yeast-to-hyphae conversion in *C. auris* strongly varies between isolates and clades. Strains from clades II and III appear to exist mostly in the yeast morphology. By contrast, pseudohyphae, which are much shorter than true filaments, are observed in approximately 84% of clade I strains [94]. *C. auris* pseudohyphae can be induced by 10% NaCl in YPD medium at 37°C and 42°C [41] or when using non-fermentable glycerol as the sole carbon source [95]. Additionally, exposure to the Hsp90 inhibitor geldanamycin [96] or the genotoxic compounds hydroxyurea and methyl methanesulfonate [97] triggers pseudohyphal growth of *C. auris*. Interestingly, pseudohyphal growth is also induced after passage in a murine host [88], yielding three distinct morphotypes. First, typical ellipsoid yeast cells rarely make filaments under *in vitro* conditions or upon exposure to typical *C. albicans* filament-inducing stimuli, including serum, Lee’s GlcNAc, and Spider agar [88]. Second, hyphal-competent (HC) yeast-form cells can enter pseudohyphal growth at high frequency following temperature induction. Third, cells form pseudohyphae at low temperatures (<25°C) but revert to HC morphotypes at 37°C, establishing a phenomenon opposite to that observed in *C. albicans* [88]. However, these findings were based on a single clinical isolate, and it remains unclear to what extent this phenotype varies cross strains and other clades.

Of note, several morphotypes, including yeast, elongated and filamentous forms as well as intermediates can coexist and emerge from a single clinical patient isolate. These distinct morphologies not only differ in cellular appearance but also in their antifungal drug susceptibilities [89]. Notably, clinical clade I to IV isolates exhibit both yeast and filaments *in vitro* when cultured on YPD phloxine B agar at 25°C. While early studies suggest that *C. auris* forms mainly pseudohyphae, subsequent evidence supports the presence of true hyphae, at least in some clinical isolates [88,89]. Importantly, morphological plasticity in *C. auris* may occur independent of host defense, implying that morphogenesis is subject to pronounced strain to strain variations. Interestingly, filamentous *C. auris* appears to increase host mortality, thus showing hypervirulence when compared to yeast-form cells [89], including elevated fungal burdens in infected tissues [88]. However, yeast-form cells secrete higher levels of secreted aspartyl proteases (SAPs) when compared to filamentous cells at 25°C. This difference disappears at 37°C, perhaps owing to the filament-to-yeast transition [88]. By contrast, the *elm1∆* filamentous mutant exhibits attenuated virulence in skin infections, likely due to the induction of a beneficial IL-17 response [98]. This difference may be explained by a dysregulation of cell wall-related genes such as the chitinase *CTS1* in *elm1∆* mutants [99]. Despite these mechanistic insights, the role of filamentation in *C. auris* pathogenesis remains uncertain. However, extensive strain-to-strain variation poses major challenges for the mechanistic dissection of morphogenesis at the molecular level, and may partly explain inconsistent or even contradicting reports. For example, low temperatures induce filamentation *in vitro* and appear to maintain filamentous growth on the skin *in vivo*. This is in sharp contrast to systemic infections, where yeast cells are most frequent in clinical isolates [88,100]. This pattern suggests that filamentation may contribute to skin tropism in *C. auris*, it is opposite to *C. albicans*, which readily filaments at human body temperatures but preferably grows in the yeast-morphotype at lower temperatures [100]. Importantly, our data show that pseudohyphae can also occur at 37°C in *ex vivo* human and mouse skin once cells reach deeper compartments, but not on the skin surface [85]. Together, these observations indicate that *C. auris* hyphal formation is not restricted to lower-temperature niches such as the skin surface, but may also contribute to tissue penetration and systemic infection [85].

**Figure 1.**
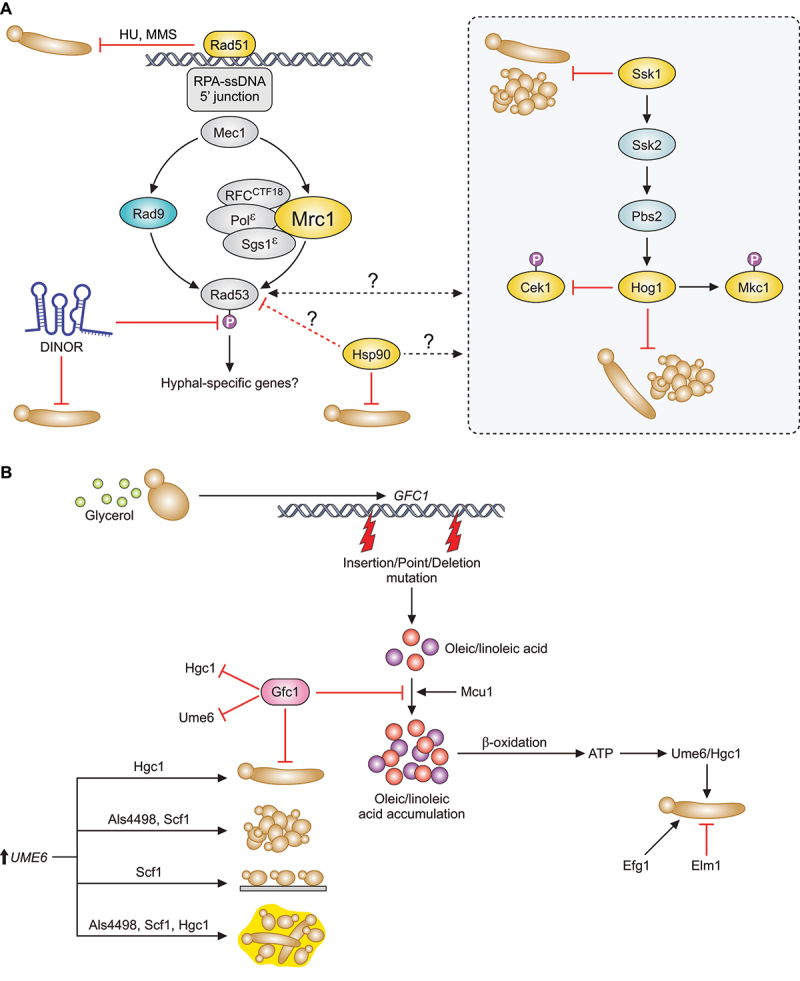
Mechanisms contributing to *Candida auris* filamentation. (A) *C. auris* can initiate filamentation in response to S-phase stressors such as methyl methanesulfonate (MMS) and hydroxyurea (HU) through activation of the Rad53 DNA damage response pathway [1]. The long non-coding RNA DINOR also modulates hyphal formation by inhibiting Rad53 phosphorylation [2]. Additionally, Hsp90 and Hog1 signaling have been implicated in promoting filamentation [3,4]. (B) Multiple signaling pathways linked to adhesion and biofilm formation regulate morphogenesis, highlighting the complex regulatory networks that shape *C. auris* pathogenicity. Illustrations were created based on conceptual frameworks [1,4,5].

Interestingly, the DNA damage response pathway has also been implicated in pseudohyphal formation in *C. auris*. Deletion of genes associated with the S-phase checkpoint control, including *RAD51, RAD9 or MRC1*, leads to abnormal cell morphology and hypersensitivity to genotoxic compounds, indicating a potential connection to pseudohyphal growth [97]. This pathway may also involve the heat shock protein Hsp90, as its deletion triggers pseudohyphal growth [96]. Additionally, the long non-coding RNA *DINOR* acts as a regulator of hyphal formation and virulence through the DNA checkpoint kinase Rad53 (Figure 1(A)) [101]. Hsp90 may also communicate with the Ssk1-Ssk2-Hog1 signaling pathway, thereby contributing to both drug resistance and hyphal regulation in *C. auris* [96,102]. Of note, the role of this cross-talk in hyphal regulation appears highly complex. For example, deletion of *SSK1* or *HOG1* in three isolates from clades I and III results in pseudohyphal formation in only one strain, again highlighting strain-to-strain variability, and suggesting the potential involvement of other genetic networks [102]. Notably, the Ssk1-Hog1 pathway inhibits Cek1 phosphorylation, while at the same time promoting Mkc1 phosphorylation. Thus, the precise mechanistic connections of Hsp90, S-phase checkpoints, and Hog1-related signaling pathways and their roles in regulating filamentation in *C. auris* remain unclear (Figure 1(A)).

Glycerol as a non-fermentable carbon source induces a rod-like filamentation-competent phenotype in *C. auris*, characterized by elongated cells appearing in long-term cultures [95]. Growth in glycerol also induces mutations in *GFC1*, an orthologue of the biofilm regulator *BCR1* in *C. albicans*. Gfc1 regulates fatty acid β-oxidation by controlling utilization of several carbon sources involving an Mcu1-dependent mechanism [95]. Moreover, this network may also involve Ume6 and Hgc1, which further sustain filamentation and elongation (Figure 1(B)) [103].

### Cell aggregation

Aggregation is a notable phenotypic trait in *C. auris*, characterized by the formation of multicellular clusters in liquid culture media or in response to environmental cues [104]. Strains from clade III usually form either large or small aggregates, while aggregation in cells from other clades appears highly strain-dependent. Most clade I strains do not naturally aggregate, but this phenotype can be reversibly induced by triazole or echinocandin treatment, whereas flucytosine and amphotericin B show no such effects [94]. Environmental conditions or growth media significantly influence aggregation traits. For example, mammalian cell culture media such as RPMI1640 suppress aggregation, whereas rich media such as Sabouraud dextrose enhance cluster formation [104]. Hence, as yet unknown media components present in highly complex media may explain inconsistencies seen in several studies. Of note, aggregation that occurs in rich media can be easily reversed by dispersing cells in pure water, yet cells re-cluster in PBS, indicating a role for salt and electrostatic forces in cell-cell interactions [104].

The relationship between aggregation and pathogenesis is complex and remains incompletely understood owing to contradictory reports [32]. Some studies suggest that aggregating strains exhibit increased resistance to disinfectants [105], azoles, and amphotericin B (AMB), potentially due to reduced drug exposure or enhanced biofilm formation [106]. Additionally, macrophages are less effective at engulfing aggregated *C. auris* clumps which may further contribute to immune evasion *in vivo* [104]. However, infection models using *Galleria mellonella, Caenorhabditis elegans* and murine systemic infection models indicate that aggregating *C. auris* phenotypes are generally less virulent than non-aggregating phenotypes [107,108], while other reports show no clear correlation between aggregation and virulence in either *Galleria* or neutropenic murine models [109,110]. These inconsistencies illustrate some of the challenges in *C. auris* research, most of them owing to extensive and extreme strain-to-strain variations. Moreover, virulence outcomes in infection models are strongly influenced by pre-infection culture conditions [111], especially for strains with moderate or low virulence, whereby *C. auris* pre-grown in complex media exhibit increased pathogenicity [104]. Overall, the collective evidence suggests that the aggregation phenotype may show attenuated virulence but is more commonly associated with skin colonization, increased biofilm formation, and enhanced environmental persistence when compared to non-aggregating isolates.

Nonetheless, aggregation traits may facilitate *C. auris* dissemination and person-to-person transmission within hospital environments [103,104]. The mechanisms underlying aggregation in *C. auris* can be categorized into three subtypes, such as cell-cell adhesion, extracellular cohesive matrix, and defects in cell separation [112]. Cell-cell adhesion-mediated aggregation engages the Als4112 adhesin [106,113], which interacts with Scf1 [114] to promote amyloid-like clustering [91]. A role for Als4112 in aggregation is further supported by the observation that thioflavin-T, an amyloid inhibitor, impairs aggregation [91]. Clustering in a large proportion of clinical isolates appears to correlate with genomic amplification of *ALS4112* [106]. NCBI sequencing data indicate that dozens of clinical isolates harbor amplified copy numbers of *ALS4112*, whereas a truncation or loss of Als4112 reduces aggregation [106]. Lack of *ALS4112* also reduces virulence in both *Galleria mellonella* [104] and in a mouse model [115], supporting a role in *C. auris* virulence. Additionally, treatments with proteinase K, trypsin, or SDS significantly reduce aggregation, suggesting that adhesins and hydrophobic interactions are pivotal when *C. auris* is encountering host defense, similar to what has been observed in members of the *C. haemulonii* complex [90]. Of note, the Ume6 transcriptional regulator contributes to aggregation, since it controls expression of both Scf1 and Als4498, another adhesin family member [103]. It is tempting to speculate that more than 13 adhesin-like proteins present in the *C. auris* proteome [113] are not simply redundant but instead, play distinct roles depending on the specific infection context or immune defense (Figure 1(B)).

The extracellular matrix (ECM) appearing during biofilm formation may also contribute to aggregation [91]. A comparison of eight *C. auris* strains, including aggregated and non-aggregated isolates by SEM, indicates that aggregating cells at 30°C are coated by a “blanket-like” ECM that is unevenly distributed in distinct areas. The ECM increases in both forms at 37°C [91]. This phenomenon might explain why both aggregative and non-aggregative clumpy isolates observed *in vitro*, can still cause damage of infected organs in murine models [113,115,116]. Furthermore, echinocandin-induced fungal clustering appears linked to defects in cell separation, as lack of genes guarding cell wall homeostasis trigger aggregation phenotypes [104]. Echinocandins destroy cell wall architecture and integrity, potentially involving the Ssk1-Hog1-Mkc1 signaling axis, which is modulated under drug-induced stress [102]. Moreover, deletion of *SSK1* or *HOG1* induces aggregation in a clade I clinical isolate [102]. However, it remains unclear whether the Hog1 pathway influences aggregation by directly affecting adhesion or cell separation defects. Although deletion of *HOG1* also results in the upregulation of Als4112, the overall impact of this adhesin on *hog1*∆ appears minimal [117].

**Figure 2.**
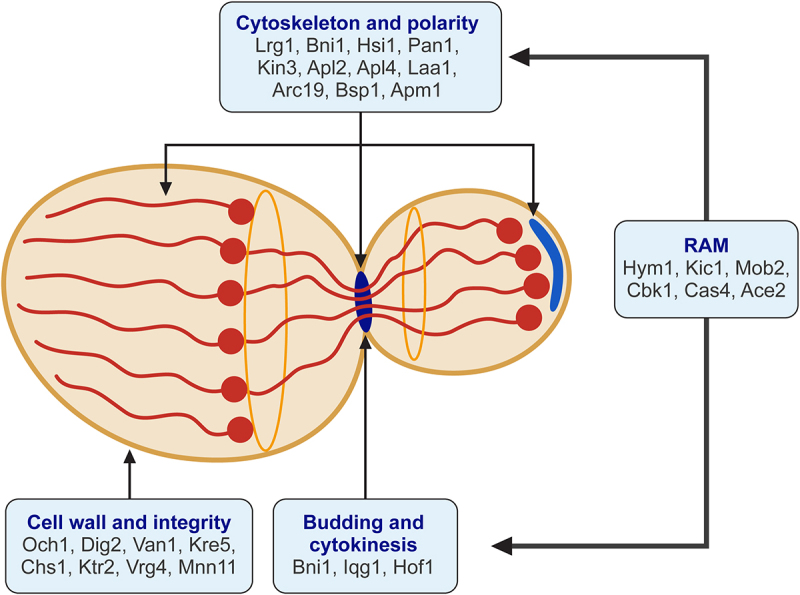
Gene families are implicated in *Candida auris* aggregation. Mutations of genes that regulate budding, cell division, polarity, and cell wall integrity trigger aggregation phenotypes. The illustration was created based on conceptual frameworks [6].

A recent *in vivo* microevolution approach uncovers additional potential mechanisms linked to cell separation defects in *C. auris* clusters [118]. Genetic ablation of chitin biosynthesis regulators (*ACE2*, *TAO3*/*CAS4*), chitin synthase (*CHS1/CHS2*), the cytoskeletal polarity protein (*BNI1*) or the GTPase activator (*LRG1*) enhances *C. auris* aggregation (Figure 2) [99,118]. Mutations in these genes are arising *de novo* during infection may potentially contribute to the *in vivo* adaptation of *C. auris* [99,118]. Indeed, those mutations exhibit fitness advantages over yeast cells in brain infections but lower pathogen burdens in other organs [118,119]. Aggregation may also attenuate virulence in some organs due to improved fungal clearance arising from enhanced neutrophil recruitment or protective IL-17–mediated inflammation, as in the case of *ace2*∆ mutants in a murine intradermal infection model [119].

### Phenotypic white-brown switching

Phenotypic White-Opaque switching is a well-characterized reversible and heritable epigenetic phenomenon in *C. albicans* [120–124]. Although reports on colony switching in *C. auris* remain limited [86,93], compelling evidence and our work demonstrate that morphogenetic switching is highly prevalent in *C. auris* [86,87,93]. A clinical strain from clade IV exhibits a three-way switching pattern on CHROMagar, displaying pink, white, and dark purple colony morphotypes [86]. The switching frequency in *C. auris* appears to be much higher than observed for White-Opaque (W/O) switching in *C. albicans* [125]. In *C. albicans*, the reversible transition between “white” and “opaque” cell states is associated with distinct virulence characteristics, mating competencies, as well as antifungal susceptibility [121]. A three-way switching model involving “white-gray-opaque” phenotypes has also been proposed [126]. Of note, the colony variations of *C. auris* on CHROMagar may resemble distinct phenotypic states identified in *C. glabrata*, which encompass white, light brown, dark brown, and very dark brown morphotypes on nutrient agar containing copper (II) sulfate or phloxine B [127]. However, in-depth investigations into *C. auris* are unavailable as yet.

Epigenetic cell-fate switching is referred to White-Opaque in *C. albicans*. Clinical observations on CHROMagar and on rich YPD agar show *C. auris* undergoes a heritable, reversible White‑Brown (W/B) conversion at high frequency, yielding colonies with distinct brown pigmentation [92,93]. Phenotypic switching in *C. auris* is stochastic and yields multiple morphotypes within a given population pool. Individual cells differ in their propensity to convert upon external cues (temperature, carbon source, stationary-phase, stress), creating a heterogeneic pool of cells that likely phenocopies the morphotypic ranges seen across clinical isolates. Temperature and carbon source are major drivers of W/B morphogenesis. In *C. auris*, glycerol (not N-acetylglucosamine) promotes W/B conversion, whereas glucose accelerates Brown-White (B/W) transition. Elevated temperature increases switching in most clinical strains. This is consistent with the skin tropism of *C. auris*, as glycerol is provided by aquaporin-3 from keratinocytes and also commonly used in skincare products. Lipases highly expressed in Brown cells may link to skin colonization by hydrolyzing host-derived triglycerides into glycerol and free fatty acids, thereby providing accessible carbon sources and potentially modulated during W/B switching.

White and Brown morphotypes display distinct stress response and drug susceptibility. Brown cells in several strains show reduced fitness under membrane, osmotic, oxidative, and cell-wall stress conditions, and display enhanced susceptibility to caspofungin, voriconazole, flucytosine, as well as to amphotericin B at higher temperatures. Derivative clones from Brown can further diverge, showing elevated tolerance to stressors, consistent with a higher mutation rate in Brown compared to White [92]. Although Brown cells exhibit enhanced adhesion, lipase activity, and SAPs, they surprisingly show impaired fitness *in vivo* with regard to skin colonization and intradermal infection. Differences in cell wall composition between White and Brown cells may explain their altered cell wall stress responses and impact immune evasion. In fact, Brown cells are phagocytosed with higher efficiency by primary macrophages.

Brown is an unstable transition state that readily switches, thus creating diversified cellular pools. Notably, many Brown-derived clones, isolated at 37°C, show reduced switching at the same temperature despite retaining lighter Brown pigmentation, suggesting stabilization of new lineages after stochastic switching under host-mimicking conditions. Accumulated mutations likely modulate pigment and switching frequency, and additional epigenetic states may contribute to the utmost complexity. Together, W/B switching plus genomic microevolution under environmental stress or clinical therapy provides a complex mechanistic basis for the extreme plasticity that modulates virulence and antifungal susceptibility of *C. auris*.

Interestingly, using transcriptomic analysis, we found more than 30 transcription factors differentially expressed between White and Brown cells. Some are conserved between *C. auris* W/B and *C. albicans* W/O switching, although their regulatory tendencies are mostly different. White and Brown cells possess distinct transcriptomes, with approximately 5.2% of genes differentially expressed between W/B, including those related to metabolic adaptations, cell-wall and membrane homeostasis, biofilm formation, and nutrient uptake, similar to the phenotypic differences observed between Brown and White cells. Notably, Wor1, Crz2 and Msn4 were among the most highly regulated, showing significant decreases of approximately 17-fold, 11-fold, and 10-fold, respectively, in Brown cells when compared to White cells. We identified these three factors as W/B activators, since deletion of each reduced the switching frequency, while overexpression caused a remarkable increase in switching, even when mRNA expression was not markedly changed. This suggests that the correlation between switching frequency and transcript levels of these regulators across clinical strains should be further investigated. Additional regulators such as Rca1 and Efg1 affect W/B switching, and they are also pivotal for the carbon-sensing pathway required for scavenging CO_2_ from ambient air [57]. *C. auris* isolates show broad variability across patients and clinical settings, posing major challenges for antifungal therapy due to treatment failures and recurrent infections. The greater its capacity to diversify its population in a given host, the higher its potential to adapt to different niches and to become refractory to antifungal therapy. Therefore, blocking *C. auris* morphogenetic conversions may improve treatment outcomes and reduce recurrence.

## Adhesion mechanisms and pathogen responses

**Figure 3.**
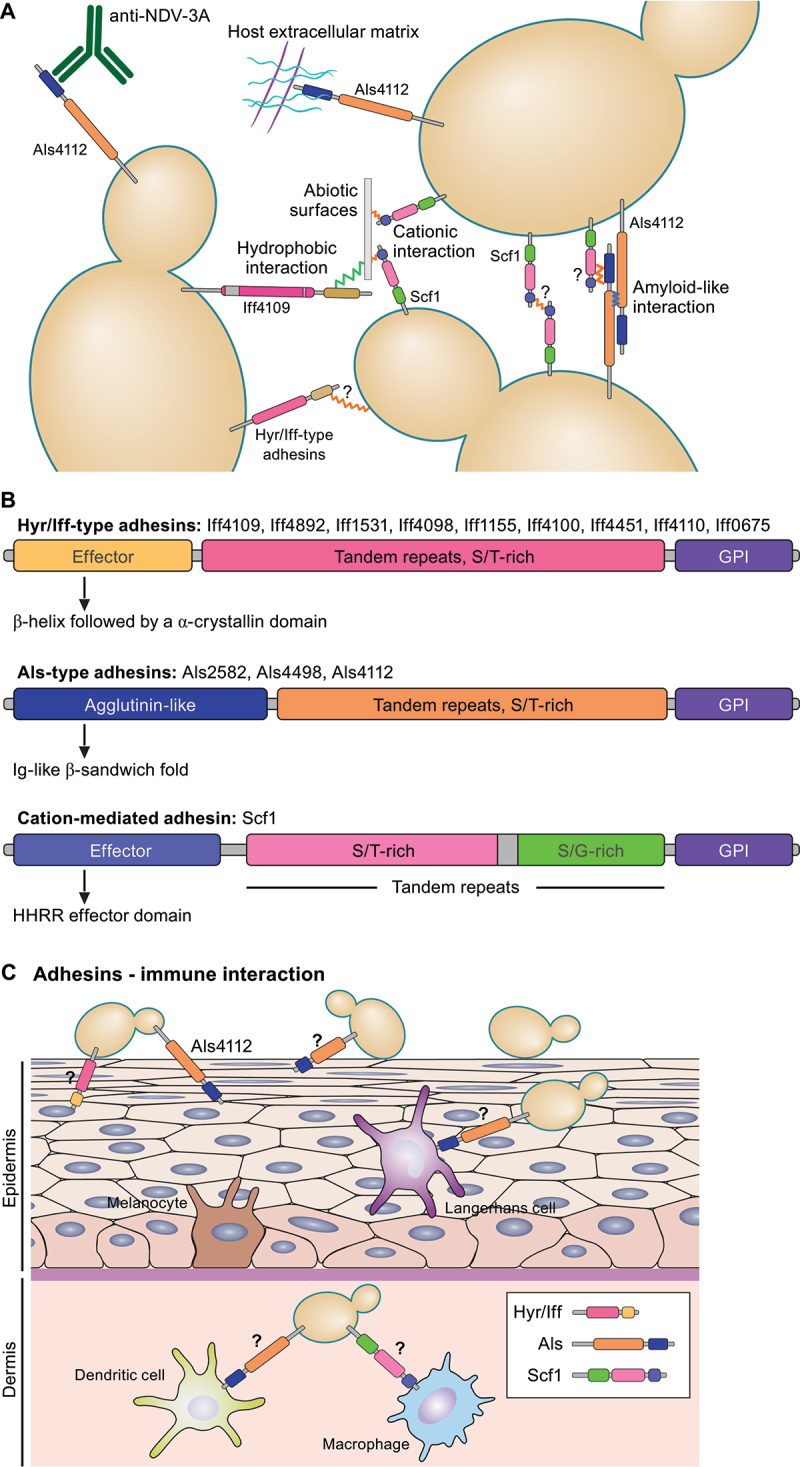
Adhesins in *Candida auris*. (A) Overview of characterized adhesin functions in *C. auris*. (B) Schematic representation of the primary domain architecture of three *C. auris* adhesin families. (C) The mechanisms underlying interactions between *C. auris* adhesins and immune cells remain poorly defined.

In general, *C. auris* exhibits lower virulence when compared to *C. albicans* [105,128]. However, *C. auris* demonstrates a remarkable ability to effectively colonize skin, but also persist on abiotic surfaces such as plastic catheters or other indwelling devices [129,130]. This persistence is key to its successful dissemination and the subsequent risk of disseminated invasive infections of immuno-compromised patients. Beyond culture-based evidence, recent metagenomic epidemiology in nursing homes further supports the skin as a major reservoir that can sustain clonal *C. auris* carriage and transmission within healthcare networks [56]. Moreover, *C. auris* can form dry-surface biofilms that enhance persistence and increase tolerance to sodium hypochlorite, providing a mechanistic explanation for long-term environmental survival despite routine disinfection [131]. Patient colonization involves not only the initial adhesion process, but also the establishment of long-term persistence on the skin surface, including biofilm formation in this nutrient-limited niche. Skin colonization likely engages metabolic interactions with resident microbial microbiome members as well as with physiological skin architecture and its distinct compartments and layers. The initial adhesion as well as dissemination of *C. auris* to deeper skin compartments is closely linked to its ability to adhere to surfaces. The genome of *C. auris* encodes 13 adhesin-like proteins, most of which are shared between clade I and III [15] (Figure 3(B)). However, clade IV lacks the putative Hyr/Iff4110 adhesin, and clade II contains only five putative adhesins due to large subtelomeric deletions [15]. This may explain why clade II isolates are clinically less relevant [15]. The functions of most *C. auris* adhesins remain largely enigmatic, particularly the Hyr/Iff-like adhesins, although they are notably enriched in the *C. auris* genome, with at least nine homologues [15,113]. The potential functional redundancy and niche-specific roles of adhesins across different environmental and host conditions asks for further investigation. Functionally, Iff4109 and the novel adhesin Scf1 are critical for the adhesion capacity of *C. auris* (Figure 3(A)). Scf1 plays a pivotal role for adhesion to skin, abiotic surfaces, biofilm formation as well as virulence *in vivo* [113]. *SCF1* transcripts are abundant in *C. auris*, engaging cation-dependent interactions for binding to negatively charged tissues such as human skin or to medical devices exposed to biological fluids. In contrast, Iff4109 exploits hydrophobic interactions, making it well-suited to interact with apolar skin lipids, sebum, or plastic polymer-based medical materials. Both Scf1 and Iff4109 cooperate to orchestrate *C. auris* adherence biotic and abiotic substrates, thereby enabling both cutaneous persistence and device-associated biofilm growth.

Als4112 from the *ALS* family adheres to host ECM proteins such as laminin and collagen V, but not to collagen I or III and its loss reduces skin colonization and biofilm formation in preclinical models [115]. The adhesion to ECM facilitates *C. auris* binding to keratinocytes and may promote surface skin colonization without triggering a significant immune response. Of note, Als4112 is also recognized by the Als3-targeting fungal vaccine NDV-3A [132], suggesting functional similarities with *C. albicans* Als3 [133]. Als3 interacts with epithelial E-cadherin, thereby inducing fungal phagocytosis [134] that engages clathrin and cortactin, along with EGFR and c-Met, all of which forming an endocytic complex engulfing *Candida* cells [134–136]. Additionally, *ALS* family members are recognized by the CD11b (domain I) receptor on macrophages, which synergizes with β-glucan-bound lectin-like domains to activate CR3 that promotes fungal clearance in systemic infections [137]. Therefore, further characterization of *C. auris* adhesins, particularly their direct and indirect interactions with immune cells, will be crucial for understanding how these proteins contribute to pathogenesis and for guiding adhesin- or surface-targeted antifungal strategies (Figure 3(C)).

After attachment, biofilm architecture and adhesin expression are shaped by coupled signaling, transcriptional, and metabolic programs [57,95,99,102,138–140]. The Hog1 MAPK influences cell-wall remodeling and immune exposure, with loss of Hog1 increasing β-glucan exposure and impairing biofilm fitness [102]. Ume6 activation strengthens adhesion and biofilm biomass and upregulates cell wall/adhesion genes (including Scf1), linking morphogenesis and surface persistence [103]. In contrast, Wor2 negatively regulates biofilm formation through modulation of *SCF1* and *ALS4112* expression, although its regulatory role appears to be clade- or strain-specific, likely reflecting underlying genotypic divergence [141]. Growth/nutrient sensing further gates biofilm development via TOR [142]. Finally, survival on nutrient-poor skin is supported by CO_2_ assimilation into central carbon metabolism via carbonic anhydrase Nce103 and downstream pyruvate carboxylase *PYC2*, a pathway that is required for robust growth on skin tissue [57]. Bicarbonate (HCO_3_^−^), generated by the carbonic anhydrase reaction, is sensed by the cAMP/PKA signaling pathway and subsequently regulates adhesion and biofilm formation via Efg1 [57]. The ability of *C. auris* to form biofilm and colonize on human skin is also supported by its capacity to utilize alternative nutrient sources, including lipid metabolism mediated by lipases [143], and to maintain cellular homeostasis, such as potassium ion balance and intracellular pH [144].

## Host-pathogen interactions at the skin barrier: immune networks against *C.* *auris*

Innate and adaptive circuits jointly shape the outcome of cutaneous *C. auris* exposure. Neutrophils, monocytes, dendritic cells (DCs), and innate lymphoid cells (ILCs) are recruited to colonized skin, yet immune defense appears attenuated when compared with *C. albicans* under similar conditions [145]. Mechanistically, this altered outcome reflects a combination of (i) skin microanatomy and immune topography, (ii) distinct fungal cell-wall architecture and morphotype-associated ligand display, and (iii) cytokine polarization that can paradoxically favor persistence rather than clearance (Table 1) [128,146].

**Table 1.** Key determinants shaping cutaneous immunity to *C. auris.*

Category	Factor	Impact on immunity	References
Fungal Factors	Morphological form (filaments & aggregation)	Aggregative and filamentous morphologies elicit stronger type 17 immune responses than yeast forms, thereby reducing fungal burden.	[98,119]
	Cell-wall mannan structure	Unique mannan layers promote immune evasion by dampening phagocyte activation and cytokine production. Their removal exposes hidden PAMPs and increases susceptibility to neutrophil-mediated killing.	[128,146,147]
	Dynamic β-glucan shielding	When exposed to environmental stressors, including low oxygen or lactate, *C. auris* masks surface β-glucans, thereby limiting macrophage recognition, reducing phagocytic uptake, and altering immune signaling.	[148]
	Surface adhesins (Scf1, Iff4109, Als4112)	Surface proteins promote epithelial adhesion and long-term skin colonization. Als4112 facilitates epithelial colonization with minimal inflammatory activation, while CD11b-mediated recognition can promote fungal clearance.	[132,137]
	Phenotypic switching	“Brown” phenotypic variants are more readily engulfed by macrophages but are less effective at sustaining stable colonization on skin surfaces.	[92]
Host Factors	Follicular microenvironment	Hair follicle persistence promotes a specialized cDC1/T-bet^+^ type 1 niche that protects *C. auris* from immune clearance.	[149]
	Epithelial barrier status	Mechanical injury (micro-wounding) facilitates deeper tissue entry, induces pseudohyphal growth, and triggers the release of inflammatory markers such as CXCL8.	[85]
	Cytokine network balance	Type 17 pathways (IL-17/IL-23) suppress fungal burden, whereas reinfection-associated IL-12/IFN-γ signaling may disrupt these protective circuits and promote persistence.	[150]
	Myeloid polarization	*C. auris* promotes IL-1Ra-dominant macrophage polarization, suppressing inflammatory signaling and impairing neutrophil-mediated antifungal activity.	[151]
	Antimicrobial peptides (AMPs)	Keratinocytes release a variety of protective peptides including hBD-3, RNase 7, and S100A7, contributing to fungal control by disrupting membrane integrity or inducing programmed cell death.	[152–158,162]
	CLR-CARD9 signaling path	Effective host defense against *C. auris* depends on CARD9-mediated signaling, as loss of this pathway results in inadequate T cell activation and cytokine production.	[159]

A key skin-specific feature is the hair follicular immune niche. Recent mapping of skin immune topography shows that Th1/Tc1 cells and cDC1 naturally line the upper hair follicle, and during *C. auris* colonization, but not *C. albicans*, this peri-follicular type 1 niche expands. In this setting, cDC1 and Tbet^+^ cells surround *C. auris* yeasts, and cDC1 extend projections between follicular epithelial cells, consistent with an anatomical “type 1” microenvironment that can foster fungal persistence [149]. Fungal morphotype also tunes these responses: filamentous and aggregative forms enhance IL-17-dominated immunity and correlate with lower fungal burdens than yeast-form cells in murine skin, supporting morphology-dependent immune activation [98,119].

At the level of innate sensing, keratinocytes and myeloid cells detect *Candida* through pattern-recognition receptors (PRRs), including Toll-like receptors and C-type lectin receptors (CLRs; Dectin-1/CLEC7A for β-glucans, Dectin-2/CLEC6A for α-mannans), activating Syk-CARD9 and other inflammatory pathways. Compared with *C. albicans*, *C. auris* exhibits distinct outer mannan layers enriched in β-1,2 linkages and unique M-α-1-phosphate side chains, features that affect CLR recognition, dampen phagocyte activation, and skew cytokine outputs [128,146,147]. In line with the central role of CLR-adaptor signaling, CARD9 has been shown to be required for effective systemic host defense against *C. auris*, with Card9 deficiency causing impaired inflammatory cytokine responses and attenuated T cell responses during infection [159].

Cell-wall architecture is also dynamic and environment-responsive, further complicating immune recognition. Moreover, recent work shows that physiologically relevant cues (e.g. lactate, hypoxia, and sublethal antifungals) can reduce β-glucan exposure in *C. auris*, leading to decreased macrophage phagocytosis and altered cytokine release, consistent with inducible immune evasion [148]. At the structural level, solid-state nuclear magnetic resonance (NMR) analyses indicate that *C. auris* and *C. albicans* share an overall cell-wall architecture but differ in adaptive remodeling responses to echinocandins. Specifically, *C. auris* maintains wall integrity via β-1,6-glucan-linked compensation rather than the thickening/chitin-glucan dynamics prominent in *C. albicans* [160]. Together, these findings support a model in which environmental and drug pressures reshape PAMP exposure and downstream sensing at the skin barrier [128,147,161].

Downstream of PRR engagement, skin-resident cells, particularly keratinocytes, deploy antimicrobial and inflammatory programs that shape early containment at the barrier. Keratinocytes produce a repertoire of antimicrobial peptides (AMPs), including human β-defensins, the cathelicidin LL-37, RNase 7, thymic stromal lymphopoietin (TSLP), and members of the S100 protein family [152–157,162]. Several β-defensins (hBD-1, hBD-2, and hBD-3) exhibit candidacidal activity [163,164], with hBD-3 shown to induce apoptotic cell death in *C. auris* under *in vitro* conditions [165]. RNase 7, which is produced at high levels by keratinocytes, contributes to broad-spectrum antimicrobial protection, including potent activity against *C. albicans*, through a combination of membrane-disruptive and ribonuclease-dependent mechanisms [155,158]. In addition, keratinocytes constitutively express the short isoform of TSLP (sfTSLP), which has been reported to exert antimicrobial effects against both Gram-positive and Gram-negative bacteria [152,166,167]. Although the antibacterial properties of many AMPs are well characterized, their direct antifungal activities remain less fully defined. Among S100 proteins, S100A7 (psoriasin), S100A8/A9 (calprotectin), and S100A12 have been implicated in antifungal defense [168], with S100A7 demonstrating direct antifungal activity against *C. albicans* [169]. In addition to AMP-mediated protection, both keratinocytes and fibroblasts produce cytokines and chemokines that coordinate inflammatory responses and immune cell recruitment. Fibroblasts, long regarded as primarily structural components, respond to *Candida* exposure by secreting IL-6 and CXCL8/IL-8, adopting antimicrobial programs that restrict dermal invasion, and inducing matrix metalloproteinases involved in tissue remodeling and repair [170–172]. Fungal infection also activates TNF signaling and ferroptosis-associated pathways within the skin [173]. Keratinocytes challenged with fungal pathogens produce pro-inflammatory mediators such as IL-1β, TNF, IL-6, and CXCL8 across multiple epithelial contexts [174–176]. Functionally, TNF serves as a neutrophil-priming factor that enhances oxidative burst capacity, whereas IL-6 primarily modulates inflammatory responses [177,178]. Collectively, these epithelial and stromal defense mechanisms establish a first line of defense against *C. auris*.

Neutrophil engagement represents another divergence point. Across diverse models, *C. auris* shows weaker neutrophil recruitment and reduced phagocytosis/killing. Its β-1,2-rich mannan displays selective binding to IgG and mannose-binding lectin and differs from *C. albicans* in phosphate decorations, together altering PRR/complement engagement [128,146,147,161]. Neutrophil extracellular trap (NET) formation contributes to antifungal defense via CLR-Syk signaling, ERK/MAPK stress pathways, and NADPH-oxidase-derived ROS inputs, with PAD4-mediated histone citrullination contributing in many settings. However, *C. auris* induces much weaker NETosis and killing, and the precise ligand-PRR circuits controlling these outcomes remain unresolved [179–184]. Immune evasion and persistence are reinforced by myeloid-state remodeling: single-cell transcriptomics of infected skin reveal an IL-1 receptor antagonist (IL-1Ra)-biased macrophage state that dampens IL-1 R signaling and suppresses neutrophil effector functions [151]. Comparative intradermal infection studies similarly highlight distinct skin immune dynamics after *C. auris* vs *C. albicans* challenge [185,186].

Host-side regulators may further gate neutrophil antifungal activity in skin. Deletion of Galectin-3 reduces susceptibility to *C. auris* skin infection by enhancing neutrophil antifungal function [187]. Consistent with the concept that myeloid function can be therapeutically “tuned”, adjunctive GM-CSF improves host defense against systemic *C. auris* infection in immunosuppressed mice [188].

On the cytokine axis, type 17 immunity is broadly protective at the skin barrier but can drive tissue pathology when dysregulated [145]. IL-17 R signaling is essential for controlling skin fungal burden, with rapid IL-17A/F release by ILCs and γδ T cells (alongside conventional αβ T cells), and infection is accompanied by rapid upregulation of antimicrobial-peptide programs by skin cells [130]. While DC-derived IL-23 is a canonical driver of Th17 differentiation, a DC-IL-23 → Th17 requirement has not yet been directly demonstrated in the *C. auris* skin model. Nonetheless, in related cutaneous settings, CD301b^+^ dermal DCs can supply IL-23 to drive early γδ T17 and later Th17 programs, whereas inflammatory macrophages/monocyte-derived DCs provide IL-12, skewing responses toward Th1/IFN-γ during *C. auris* reinfection [150,189]. In this reinfection setting, excess IFN-γ antagonizes IL-17 circuits and exacerbates disease [150]. Consistent with spatial control of polarization, the follicular cDC1/Th1-Tc1 niche expands during *C. auris* colonization and is linked to persistence [149]. Overall, a divergent T cell polarization emerges as a hallmark: secondary *C. auris* skin infection is dominated by an IL-12-driven Th1/IFN-γ program that attenuates the otherwise protective IL-23/IL-17 axis central to *C. albicans* control [150,189,190].

Finally, fungal virulence traits and cell organization modulate the quality of cutaneous immunity. In intradermal models, filamentous *C. auris* is linked to lower fungal burdens and robust IL-17 programs in ILC3s, γδ T cells, and Th17 cells, whereas yeast morphotypes dampen protective IL-17 and favor persistence. Aggregation (e.g. via *ACE2* loss) can boost neutrophil influx and IL-17-dominated immunity, lowering fungal burden on skin [119]. A single-gene mutation can rewire metabolism and unlock filamentous competence, enhancing skin colonization as highlighted by *GFC1* (*BCR1*)-linked filamentation in *C. auris* [95]. Disrupting mannosylation (*PMR1*, *VAN1*) unmasks PAMPs, enhances neutrophil killing, and can shift DC/T cell programming along the Th1↔Th17 axis [147,150,161,191]. Notably, IL-17 dependence can be context-specific, being robust in many intradermal models yet dispensable in at least one epicutaneous reinfection model, highlighting that skin site, barrier state, and inoculation route can alter which effector modules dominate [98].

Taken together, outcomes hinge on the balance between CLR-Syk-driven innate programs (including neutrophil recruitment/activation and NET-associated pathways) and DC-guided polarization of IL-23/IL-17 vs IL-12/IFN-γ circuits. Defining the *C. auris* ligands and PRRs that orchestrate these responses, and leveraging single-cell and spatial profiling, should reveal actionable targets for immunomodulation and efficient decolonization [128,146,191]. In parallel, antibody-based approaches are gaining traction: broadly reactive monoclonal antibodies that bind conserved *Candida* cell-wall epitopes can disrupt *Candida* biofilms, promote opsonophagocytosis, and improve outcomes against *C. auris in vivo* [192], aligning with broader recent syntheses of host-pathogen interaction and translational priorities [80].

## Experimental models to delineate mechanisms of *C.* *auris* pathophysiology

### *In vitro* models

Since its emergence, *C. auris* research has advanced the field from population readouts to physiologically more relevant *in vitro* and *ex vivo* systems that better recapitulate skin tropism. We discuss these platforms, including *ex vivo* human, porcine and mouse skin explants, biofilm and co-culture models as well as quantitative approaches such as genome-scale metabolic models. The common goal of these model systems is to establish controlled settings to better dissect *C. auris* biology, virulence, pathogenicity traits as well as the dynamics of complex host-pathogen interactions [85,129,193].

Various experimental models have emerged in recent years. Most efforts include comparisons of *C. auris* with *C. albicans*, revealing that *C. auris* is generally less virulent than the pleiomorphic pathogen *C. albicans*. However, *C. auris* is exceptionally well equipped to efficiently colonize skin surfaces and to form biofilms on biotic and abiotic substrates, all in all sustaining persistence in healthcare environments [128]. Given its marked skin tropism, adhesion and biofilm formation on healthcare devices as well as on skin tissues play critical roles in *C. auris* virulence. These traits are studied using *in vitro* biofilm models, by confocal or electron microscopy, along with conventional biochemical assays such as XTT and MTT exclusion, resazurin staining, and biomass quantification [129,194]. Of note, skin-mimicking synthetic sweat medium enables *C. auris* to form robust biofilms that remain viable even after extended desiccation, surpassing those of *C. albicans* [129].

The initial adherence to surface is a crucial step, as it facilitates and allows for *C. auris* transmission in healthcare settings. The Scf1 adhesin, present in *C. auris* and *C. lusitaniae* but absent in other *Candida* spp., is required for surface colonization. A fluorescent bead-based assay combined with flow cytometry can be used to quantify the adhesion capacity [113,195]. Additionally, *in vivo* skin colonization engages the Als4112 adhesin that plays a significant role in adherence to human keratinocytes [115]. A high-throughput imaging-based method to monitor adherence of about 1,500 insertion mutants, reveals that Als4112 recognizes the extracellular matrix of host cells, but does not contribute to adhesion to plastic [115].

Macrophage and epithelial co-cultures show that *C. auris* elicits weaker pro-inflammatory responses, explaining a limited neutrophil-mediated killing when compared to *C. albicans* [146,184]. Mechanistic studies show that *C. auris* takes advantage of metabolic strategies to escape phagocyte killing. *C. auris* can trigger macrophage death exploiting glucose starvation while limiting inflammasome (NLRP3) activation at the same time [196]. Multi-omics and genetic analyses map adhesin-driven, phenotype-dependent programs (biofilm/aggregative) that modulate host interactions [91,114,197,198]. Murine models corroborate these findings, showing that *C. auris* tissue tropism phenotypes elicit distinct immune responses when compared to *C. albicans* [199].

### *Ex vivo* skin models

A major advance was the development of *ex vivo* skin platforms that mimic key aspects of the cutaneous host niche environment. These models demonstrated extensive *C. auris* biofilms on porcine as well as native human and mouse skin [85,129,193]. Standardized human skin explants enable quantification of adherence, colonization, growth, and biofilm formation. Importantly, these models also allow antiseptic efficacy to be tested, providing a platform to evaluate interactions of *C. auris* with skin and medical devices. Native human and mouse *ex vivo* systems are also used to evaluate the barrier invasion capacity. In intact human epidermis, *C. auris* shows very limited penetration, whereas micro-wounding such as by needling or epidermal removal allows for dermal access. Human explant models typically mount CXCL-8 responses, indicating an active host-pathogen interaction and skin immune defense. By contrast, the unwounded mouse skin barrier is comparatively permissive for *C. auris* penetration [85]. These systems are also uncovering cell-type-specific immune responses during colonization [85,193]. Although these models markedly improve biological relevance, the limited explant viability *ex vivo* and the lack of vascularization that causes incomplete immune complexity call for optimization to further enhance their translational value. *Ex vivo* human and porcine skin closely recapitulate the stratum corneum and lipid/salt environment at the barrier that govern *C. auris* adhesion and the resulting biofilm architecture. In synthetic sweat medium as well as on native skin, *C. auris* forms dense, matrix-rich biofilms [129,193]. These platforms offer relevant models for testing topical decolonization, enabling microscopy-based assessment of lead agents, since their efficacy on tissue can differ markedly from plastic [193]. Recent *ex vivo* studies on intact and wounded human skin show that octenidine-based antiseptics can markedly reduce *C. auris* colonization, supporting octenidine benchmarking before *in vivo* evaluation [66].

Barrier integrity is pivotal, since it determines susceptibility to *C. auris* invasion. On intact adult human skin, *C. auris* remains superficial and appears unable to penetrate, often growing within skin folds and surface irregularities. However, micro-injuries permit dermal entry of *C. auris*, accompanied by a shift to pseudohyphal growth morphologies. Invasion is much less pronounced when compared to *C. albicans*, which can efficiently penetrate even intact epidermis. By contrast, intact mouse skin is comparatively vulnerable to *C. auris* invasion, likely owing to the thinner epidermis and highly abundant hair follicle shafts that can serve as entry portals. Of note, morphogenetic transitions are observed in deeper dermal layers of mouse skin, implying translational implications of murine data [85]. Although lacking the dynamics of systemic immune defense, *ex vivo* skin models facilitate resolving niche-specific host-pathogen interactions and topical pharmacodynamics, offering synergy of human and murine model systems for mechanistic aspects and validation.

To overcome limitations of *ex vivo* skin models, including restricted tissue viability and donor variability, skin organoid and skin-on-chip platforms may provide promising future tools to study *C. auris* colonization, barrier invasion, host responses, and antifungal efficacy. Organoid-based approaches are beginning to emerge in medical mycology, as illustrated by recent work using human brain organoids to study fungal infection, including *C. auris* [200]. The development of such human-relevant models is also timely in light of current FDA efforts to promote New Approach Methodologies, including skin equivalents, organoids, and organ-on-chip systems, to reduce reliance on animal testing in drug development [200–203].

### Non-mammalian *in* *vivo* models

Non-mammalian model systems (*Galleria mellonella*, *Drosophila melanogaster*, *Caenorhabditis elegans*) provide cost-effective options for testing virulence and antifungal efficacy [197,204,205]. However, the overall biological relevance is sometimes limited, demanding careful validation of data. In *Drosophila*, *C. auris* shows strain-dependent pathogenicity but enables evaluation of azole treatment responses [204]. Although the systems are amenable to high-throughput screening, they cannot fully recapitulate the complexity of human skin immune defense, making translation a significant challenge. In biofilm models that mimic wound- and device-associated infections, mixed microbial communities can substantially modulate *C. auris* behavior, the host inflammatory response, as well as the efficacy of antiseptics or antimicrobials. As most studies focus on *C. albicans*, there is a need for complex co-cultures reflecting the microbial community context and *C. auris-*specific standardized systems [206].

*Galleria mellonella* and other invertebrate models also offer cost-efficient high-throughput potential without ethical concerns. These models are employed to study virulence and to test anti-fungal synergy, enabling detection of relevant differences across isolates and morphotypes [108,109,197]. Recent datasets integrate aggregation phenotypes with antifungal MICs to predict clinical outcomes, strengthening a potential role for early triaging before engaging mechanistic studies in native skin or validation in murine models [207]. *Caenorhabditis elegans* models are also used to assess fungal survival and virulence, although *C. auris* data remain limited when compared to other *Candida* spp. [108]. Interestingly, a study using the *Aphanius dispar* killifish embryo yolk-sac microinjection model [208], suggests an enrichment of multiple xenosiderophore transporters across five *C. auris* clades, confirming the notion that iron homeostasis may be a promising antifungal target in *C. auris* [209–211].

### *In vivo* mouse models

To assess biofilm formation in medical devices, catheter models in mice and rats are available. The data reveal interactions between Scf1 and Als4112 during *C. auris* adhesion [114]. To mimic *C. auris* skin tropism, various skin colonization models are available, including long-term skin colonization [130], neonatal mouse skin colonization [95], intradermal infection models [185], as well as primary human skin models [85]. While *C. auris* can stably colonize mouse ear and back skin, *C. albicans* fails to maintain a long-term colonization [130]. Of note, colonization capacity varies across clades, with Clade III and IV isolates exhibiting much stronger skin colonization [130]. Intradermal mouse infection models further demonstrate that *C. auris* can cross the skin barrier to reach organs in both immunocompetent and immunocompromised animals [186]. Hence, *C. auris* can transition from skin infections to systemic infections although a pronounced strain dependency is evident. Further validation is thus required, also because mice have a very thin dermal layer, which may introduce confounding effects when using intradermal injection models. To address these limitations, we have established a standardized needling model [85], which generates multiple microinjuries of selectable and reproducible depth, providing a better approach for assessing skin penetration ability.

Cutaneous and intradermal models demonstrate that IL-17-driven responses protect against persistent colonization, while barrier integrity and the resident microbiota can shape outcomes [85,130,186]. A new aspect to *C. auris* skin biology has emerged, since the fungus displays a pronounced hair-follicle tropism in mice, binds human hair and concentrates at follicular orifices [149].

Integrating complementary models is necessary to decode host responses underlying *C. auris* skin persistence. Progress will likely come from deliberately combining high-fidelity barrier models (*ex vivo* native human/porcine skin) with immunity-competent *in vivo* systems (murine cutaneous and systemic models), while using non-mammalian platforms for rapid, low-cost prioritization of virulence and drug-response phenotypes. A key near-term need is methodological standardization in native-skin systems (skin source/age, barrier-injury paradigms, inocula, strain handling, and readouts), paired with explicit follicular-niche endpoints and transparent data sharing for imaging and transcriptomics [85,193].

## Knowledge gaps in *C.* *auris* pathobiology

### Methodological challenges and emerging tools in *C.* *auris* research

Over the last 15 years, significant advances have been made in understanding *C. auris* pathophysiology, including the discovery of pathways involved in adhesion, skin colonization, biofilm formation, and antifungal drug resistance [11,80]. The functional genomics toolkit for *C. auris* has progressively expanded over time, and integrated omics approaches have significantly advanced fungal research, particularly when combined with *in vivo* infection models. These strategies have enabled the systematic identification of virulence determinants and host-pathogen interaction networks [114]. For example, the integration of genomics, transcriptomics, and proteomics has revealed key virulence-associated factors and pathways, including the oxidative stress-associated peroxiredoxin Tsa1b and carbon-sensing mechanisms in *C. auris* [57,212]. Furthermore, specialized proteomic approaches, including immunoproteomics, provide additional resolution for mapping host-pathogen interactions and identifying novel antigenic targets [107].

Beyond omics-based approaches, forward genetics and phenomics methods have emerged as powerful tools for functional genomic analysis of *C. auris*. Techniques for functional genomic screening such as piggyBac transposon mutagenesis and *Agrobacterium tumefaciens*-mediated (AtMT) insertional mutagenesis have been successfully applied to identify genes and pathways involved in stress responses, morphogenesis, drug resistance as well as virulence in *C. auris* [99,101]. Additionally, approaches such as Tn-seq are being developed in other species such as *Candida glabrata* [213,214]. Recent CRISPR-based screening in *C. albicans* across diverse environments provides a practical blueprint for identifying dosage-sensitive and context-specific vulnerabilities that could be adapted for *C. auris* [215].

Single-cell omics approaches (e.g. DNA-seq, RNA-seq, and proteomics) are widely applied to dissect host responses, yet their implementation from the pathogen perspective remains limited [216]. Emerging single-cell methodologies developed for yeast and bacteria may accelerate progress in *C. auris* research [217,218]. Notably, dual scRNA-seq or multimodal single-cell readouts represent promising strategies to capture a more integrated view of host-fungal interactions, moving beyond the current compartmentalized analyses [219]. In parallel, CRISPR-based perturbation platforms [215] coupled with high-throughput single-cell profiling should be established for *Candida* spp. to enable systematic dissection of regulatory networks and gene-function relationships at cellular resolution. Finally, advanced single-cell optical imaging approaches may further enhance understanding of *C. auris* biology, particularly with respect to dynamic morphogenetic transitions under environmental stress [220]. Overall, continued investment in cutting-edge technologies will be essential to investigate pathogen biology in conditions that more closely approximate native host environments.

Beyond high-throughput readouts, functional genomic validation in *C. auris* requires more precise genome-editing strategies. The high frequency of undesired non-homologous end-joining (NHEJ) events in this pathogen poses a major limitation, often resulting in imprecise integrations and complicating mutant generation [221]. Improved tools that favor homologous recombination or enable controlled repair outcomes will be critical for robust genotype-phenotype analyses. Recent advances in episomal, Cas9-based editing enable precise genome manipulation across multiple *C. auris* clades and help causal validation of resistance and fitness determinants. However, systematic benchmarking also highlights strain dependence and variable cassette integration, underscoring the need for standardized, community-adopted editing workflows and appropriate controls [221]. Recent CRISPR/Cas9-based short homology–directed genome engineering in *Cryptococcus neoformans* achieved near-100% editing efficiency and may provide a powerful framework for improving precise genome editing in *C. auris* and other fungal pathogens [222].

### Unresolved questions in *C.* *auris* pathobiology and host immunity

During the last decades, several longstanding questions in *C. auris* pathogenesis have been addressed such as multi-drug resistance [57,77,81,223,224], adhesion and biofilm formation [95,99,103,106,113,118], and skin tropisms [56,57,115,130,149]. However, knowledge about the function of many proteins or non-coding RNAs is still missing and progress will increasingly depend on scalable, clade-aware perturbation genetics that works in host-relevant environments. Functional genomics in *C. auris* should be conducted in parallel with closely related species of the *C. haemulonii* complex, as their shared multidrug resistance may reflect conserved genetic determinants [90,225,226], while lineage-specific factors unique to this complex (yet absent in other *Candida* pathogens) could underlie their common phenotypic traits. Indeed, several *C. auris*–specific proteins, including Scf1 [113] and Rba1 [227], have been linked to distinct biological functions, underscoring the role of species-specific determinants in *C. auris* adaptation and pathogenicity.

Recent insights into epigenetic switching in *C. auris* are intriguing and may explain phenotypic variability across and within clades [92]; however, fundamental questions remain regarding how distinct cell states are established and their roles in transmission, skin colonization, and infection. Nutrient sensing warrants particular attention [196,210], as emerging links between carbon sensing and central carbon [57] and lipid metabolism [143] may underlie adaptation to skin niches, potentially coupling skin tropism with enhanced stress and antifungal tolerance. Comparative analyses of fermentative vs oxidative metabolism across *Candida* spp. may therefore help define metabolic traits that are uniquely associated with *C. auris* [57]. The persistence of *C. auris* on human skin also underscores opportunities for further investigation into interspecies interactions between *C. auris* and other members of the skin microbiome [57,58].

From the host perspective, several unresolved key questions remain, which are summarized in Table 2. A systematic comparison of the IFN-γ-skewed follicular niche vs IL-17-dominated protection would clarify reinfection biology and refine decolonization strategies [85,117,130,149,230–232]. Here, emerging single-cell profiling indicates that *C. auris* can attenuate IL-1 R-dependent defense via IL-1Ra induction and a mannan-mediated evasion mechanism, nominating the IL-1 axis as a tractable host-directed target to complement antifungals in skin [151]. Along the same lines, host immunomodulation is becoming experimentally actionable: CARD9-dependent CLR signaling is required for systemic host defense (implicating CLR–CARD9 pathway integrity as a determinant of outcome), and adjunctive GM-CSF can improve survival and reduce tissue burden in immunosuppressed mice [159,188]. Finally, readouts from spatial multi-omics approaches integrated into AI tools would help elucidate the complexity and dynamics of immune defense against *C. auris* and other fungal pathogens at both tissue and systemic levels [233].

**Table 2.** Remaining questions in host-*C. auris* interactions.

Question	Explanation	References
Which skin microniches matter most in humans?	Which compartments (stratum corneum vs upper follicle vs deeper follicle) are necessary and/or sufficient for long-term persistence and reseeding after apparent clearance?	[85,149,193]
What is the causal immune logic of “protective vs permissive” programs?	Under what conditions does IL-17-dependent protection dominate, and when do IFN-γ-skewed responses (particularly in follicular contexts) suppress IL-17 and favor persistence, potentially explaining reinfection and decolonization failure?	[130,149,150]
How does fungal morphology reprogram host responses?	Which host pathways sense and respond to heritable morphotype switching or filamentation after mammalian passage, and is the lower fungal burden associated with stronger IL-17 responses truly morphology-driven?	[88,89,98]
How do stress-response circuits couple environmental fitness to immune evasion?	Beyond general stress tolerance, what niche-specific mechanisms enable Hog1 MAPK-mediated colonization and intradermal persistence, and which host pressures (osmotic, oxidative, antimicrobial peptides) are most decisive *in vivo*?	[117,228]
Which readouts best predict clinically relevant persistence?	When do CFU, qPCR, histology/immunofluorescence, and RNA-seq diverge, and which composite endpoints (including follicular occupancy and inflammatory signatures) are most reproducible across laboratories and skin sources?	[85]
What explains isolate-to-isolate heterogeneity in host interaction?	Can multivariable isolate features robustly predict virulence, inflammatory polarization, and risk of treatment failure across model systems?	[108,109,197,207,229]
How should tissue-level antifungal response be quantified beyond static MICs?	Given echinocandin tolerance and heteroresistance signals, which dynamic tissue-based kill or clearance assays (*ex vivo* skin and murine models) best capture failure risk and inform regimen design?	[230]
Which adjunct targets meaningfully reshape host-pathogen balance in skin?	Do chromatin regulators such as Gcn5 primarily reduce intrinsic virulence, enhance antifungal activity, or both, and are these effects preserved in native skin niches?	[231,232]
What is the most efficient model pipeline for discovery-to-translation?	A rank-order strategy: (i) *ex vivo* native human or porcine skin to define niche mechanisms, imaging phenotypes, and topical interventions; followed by (ii) murine cutaneous or systemic models to integrate immunity, persistence, and dissemination, with standardized barrier injury and harmonized follicular endpoints.	[85,115,116,193,234,235]

## Data Availability

There is no data associated with this research.
